# PTX3/NF-κB/TLR4 Pathway Evaluation in the Follicular Fluid to Successfully Predict Blastocyst Implantation: A Pilot Study

**DOI:** 10.3390/biomedicines13051071

**Published:** 2025-04-28

**Authors:** Alessio Ardizzone, Carmelo Liuzzo, Arianna Ferro, Marco Galletta, Emanuela Esposito, Anna Paola Capra

**Affiliations:** 1 Department of Chemical, Biological, Pharmaceutical and Environmental Sciences, University of Messina, Viale Ferdinando Stagno d’Alcontres, 98166 Messina, Italy; aleardizzone@unime.it (A.A.); annapaola.capra@unime.it (A.P.C.); 2 UOSD Center for Medically Assisted Procreation (MAP), AO “Papardo” Hospital, Contrada Papardo, 98158 Messina, Italy; carmeloliuzzo@papardo.it (C.L.); ariannaferro@aopapardo.it (A.F.); marcogalletta@aopapardo.it (M.G.); 3 Genetics and Pharmacogenetics Unit, “Gaetano Martino” University Hospital, Via Consolare Valeria 1, 98125 Messina, Italy

**Keywords:** Pentraxin 3 (PTX3), artificial reproductive treatments (ARTs), follicular fluid, inflammation, pregnancy outcome, blastocyst implantation

## Abstract

**Background:** The implantation process is complex and involves numerous factors that can affect its success. In artificial reproductive treatments (ARTs), chronic inflammation seems to be associated with implantation failure, largely contributing to reproductive dysfunction. Pentraxin 3 (PTX3) is overexpressed in several pathological conditions by exerting a pivotal role both as a regulator and indicator of inflammatory response. Some literature data have shown that PTX3 could have an impact on follicle growth and development, influencing women’s fertility. This study aimed to detect PTX3 in follicular fluids collected during an ART protocol in relation to implantation outcomes. **Methods**: The PTX3/NF-kB/TLR4 pathway and other cytokines were assessed in the follicular fluid of 169 subjects, under the age of 40 years, undergoing IVF cycles, including females without achieved implantation (*n* = 98) and those with implantation (*n* = 71). Furthermore, subgroup analyses were performed to evaluate PTX3 values according to age difference. **Results**: From our data, PTX3 emerged as a strong predictor, more than TNFα and IL-1β, of implantation failure and related inflammatory follicular state. Overall, the results point to PTX3 as a potential biomarker for ART success, and their testing may be helpful in women whose successful implantation remains unexplained. **Conclusions**: Therefore, PTX3 could constitute a reliable biomarker and a valuable target to improve ART outcomes.

## 1. Introduction

The reproductive process comprises a series of complex biological events among which implantation is one of the most critical. The lack of implantation after the transfer of embryos—implantation failure, also repeated failures that can occur, in this case considered as recurrent (RIF)—affect approximately 10% of couples undergoing in vitro fertilization (IVF) [1]. Implantation failure must take into consideration several variables, so it represents one of the greatest challenges in the field of assisted reproduction.

Even if the process of implantation is still not completely understood for sure, a competent blastocyst and a receptive endometrium are crucial for its success, especially in artificial reproductive treatments (ARTs) [1]. This scenario could be very useful to look at potential biomarkers for implantation success in ARTs [1].

Pentraxin 3 (PTX3) is an evolutionarily conserved glycoprotein that belongs to the superfamily of pentraxin, with a crucial role in the identification of self- and non-self-antigens. Similarly to the short pentraxin C-reactive protein (CRP), PTX3 is an essential component of humoral innate immunity involved in resistance to selected pathogens and regulation of inflammation [2].

The *PTX3* gene is located in 3q25.32 and composed of three exons, with the first and second coding the signal peptide and the N-terminal domain and the third exon coding the C-terminal domain showing homology with the classical CRP and serum amyloid P component (SAP) [3].

The primary sequence of PTX3 is highly conserved among species and consists of an assembled octameric complex [3].

The expression, localization, and molecular interactions of PTX3 in female fertility have been studied in the last 10 years, demonstrating that this protein is expressed at specific sites and times during the ovarian cycle and plays different roles [3].

In detail, mouse cumulus cells during cumulus expansion produce PTX3 that localizes in the matrix. Confirming this, the PTX3^–/–^ mouse model shows a severe defect in female fertility associated with abnormalities of the cumulus oophorus and failure of in vivo oocyte fertilization [4].

PTX3 is also expressed in the human periovulatory cumulus oophorus, and it is present in the cumulus matrix as well [4], having a role in cumulus expansion. This molecule co-localizes with hyaluronic acid throughout the cumulus matrix with the help of tumor necrosis factor a-induced protein 6 (TNFαIP6), and it is involved in stabilizing the structural integrity of the cumulus oocytes complex [4].

Matured oocytes without cumulus expansion have limited implantation potential. Therefore, gene expression levels in the cumulus cells act as a mirror of the true oocyte developmental potential, but to date, there is no single gene expression that can be used to predict the implantation rate and lead to a live birth [5].

Until now, several sources of evidence have been collected to study the expression of PTX3 by human cumuli oophori. Northern blot analysis revealed that cumulus cells obtained from patients undergoing ART express PTX3 mRNA. Furthermore, the PTX3 protein could be detected by an enzyme-linked immunosorbent assay (ELISA) in the follicular fluid (mean 11.4 ng/mL, range 3.2–27.9 ng/mL) and by western blotting in the cumulus matrix [4].

Zhang et al. found a relative abundance of PTX3 mRNA in cumulus cells from oocytes that developed into embryos with higher implantation potential [6]; in contrast, some evidence failed to prove a significant association [7,8].

Thus, it is still not clear if in humans altered PTX3 systemic levels, determined by genetic background and/or low-grade chronic inflammation, can also impact the blastocyst implantation rate during ART procedures [3]. A possible role of PTX3 as a biomarker for blastocyst implantation can reflect the exact necessary balance of immunological reactions and vascular changes necessary for adequate implantation.

Previously, Freis et al. tested the serum levels of PTX3 at the time of embryo transfer between women with an ongoing pregnancy compared to those without implantation. Patients with no implantation presented significantly higher serum levels of PTX3 compared to women who became pregnant [1].

Despite this evidence, systemic PTX3 levels in women’s serum can be influenced by several factors linked to healthy status, habits, and lifestyles. Here, to provide the most accurate analysis, not influenced by confounders, we tested it in collected follicular fluids.

Follicular fluid is a serum transudate which contains metabolism products by granulosa and theca cells [9]. Follicular fluid reflects the follicular microenvironment surrounding the oocyte and is associated with follicle development and oocyte competence. Its composition consists of a variety of lipids, microRNA, polysaccharides, cytokines, growth factors, inflammatory factors, reactive oxygen species, and antioxidant enzymes [10].

Follicular fluid cells are isolated from follicle aspirates at oocyte retrieval from ART patients and have been described as a heterogeneous cell population that includes immune cells, steroidogenic cells, epithelial cells, and ovarian structural cells [9].

Our pilot study aims to detect PTX3 protein levels and PTX3’s related pathway in the follicular fluid of women undergoing IVF cycles, comparing two subgroups: females without implantation and those with successful implantation.

## 2. Methods

### 2.1. Participant Data

We reviewed the clinical characteristics of females referred to fertility counseling at the Centre for Reproductive Medicine, Papardo Hospital in Messina, between 2023 and 2024.

In total, 169 patients who were undergoing IVF treatment and from which follicular fluids were available, were selected for this study. Electronic medical records and clinic schedules were evaluated to collect all the fertility-related data. Each participant signed informed consent for all the procedures for data collection and analysis for research purposes. The group included infertile women, aged between 23 and 39 years, who underwent IVF treatment due to male factor, tubal factor, and endometrial factor and had serum AMH ≥ 1.5, 8 ≤ AFC ≤ 24, and FSH < 10 IU/L.

Exclusion criteria were females with ovarian surgery, polycystic ovarian syndrome, and endometriosis, as well as others affected by organic diseases, concomitant infections, other complications, or prolonged therapy.

All the women never had a history of pregnancy. Patients enrolled in our study received a standardized gonadotropin-releasing hormone (GnRH) antagonist. When one or more follicles developed with diameters up to 20 mm, human chorionic gonadotropin (hCG) was injected to induce follicular maturation, and eggs were received by ultrasound-guided puncture 36 h later. IVF or intracytoplasmic sperm injection (ICSI) was performed based on the semen quality and previous couples’ reproductive history. The sample totaling 169 subjects was divided into two groups: women without successful implantation (*n* = 98) and women with positive implantation outcomes (*n* = 71).

Fertilization was confirmed at 18–20 h after insemination by the presence of two pronuclei. Cleaved embryos were observed on day 3 after insemination. Blastocyst morphological assessment was carried out on day 5 or day 6 by the development of cavity, trophectoderm, and inner cell mass.

Two weeks after the embryo transfer, the hCG level in the blood was tested to determine the pregnancy, confirmed by transvaginal ultrasound after 7 to 8 weeks of pregnancy detecting a heartbeat.

For subgroup analyses, the choice to define the age cutoff at <30 years was based on established evidence in reproductive medicine, which suggests that fertility parameters, such as ovarian reserve and response to assisted reproductive techniques, begin to significantly change around the age of 30 [11]. This age threshold is therefore widely accepted in the literature as a standard for distinguishing between younger and older women in fertility research [12].

### 2.2. ELISA Kit

ELISA kits were used to measure the levels of PTX3 (Invitrogen, Thermo Fisher Scientific, CA, USA; #EH386RB; assay range: 0.08–20 ng/mL), TLR4 (Invitrogen, Thermo Fisher Scientific, CA, USA; #EH460RB; assay range: 0.4–100 ng/mL), IκBα (Invitrogen, Thermo Fisher Scientific, CA, USA; #EH253RB; assay range: 0.65–150 ng/mL), NF-κB (MyBiosource, San Diego, CA, USA; #MBS260718; assay range: 0.156–10 ng/mL), TNFα (Invitrogen, Thermo Fisher Scientific, CA, USA; #BMS223INST; assay range: 7.8–500 pg/mL), IL-1β (Invitrogen, Thermo Fisher Scientific, CA, USA; #BMS224-2; assay range: 3.9–250 pg/mL), and IL-6 (Invitrogen, Thermo Fisher Scientific, CA, USA; #BMS213-2; assay range: 1.56–100 pg/mL) in human follicular fluids. The ELISA kit was performed following the manufacturer’s instructions [13]. To corroborate our findings and rule out non-specific binding and false positive results, we added negative control samples in each plate of the ELISA tests. In general, the following protocol was used for the ELISA method, with specific variations based on what is reported in the manual of each kit.

To create a dilution series and a standard curve, we first diluted the standard solution. Next, we filled curve wells with 100 μL of the corresponding standards and the other wells with 100 μL of diluted samples. After that, the plate was covered and gently shaken while it was incubated at room temperature for 2.5 h. Following the removal of the solution and four rounds of washing with 1X wash buffer, 100 μL of the produced biotin conjugate was added to each well, and the plate was gently shaken and left to incubate at room temperature for 1 h. Then, four washing cycles were carried out again. After that, each well received 100 μL of the prepared Streptavidin-HRP solution, and the plate was gently shaken and allowed to incubate for a further 45 min at room temperature. Again, the four washing cycles were performed. Each well received 100 μL of TMB substrate, which caused the substrate to become blue. In this phase, the plate was gently shaken while it was incubated for 30 min at room temperature in the dark. After adding 50 μL of Stop Solution to each well, the solution in the well became yellow. We next used a microplate reader (Thermo ScientificTM MultiskanTM FC Microplate Photometer, Waltham, MA, USA) to measure the absorbance at 450 nm.

### 2.3. Statistical Analysis

The values are presented as the mean ± SD of N observations, where N represents the number of cases included in the study. First, variables between groups were assessed using t test. Obtained data were first analyzed to assess normality; based on the results that indicated a non-normal distribution of data or a small sample size, the Mann–Whitney test was used to evaluate statistical significance by employing GraphPad version 8.0 (La Jolla, CA, USA). Only findings with a *p*-value less than 0.05 were considered significant. Additionally, Pearson correlation analyses were performed to assess the relationships between variables.

## 3. Results

### 3.1. Preliminary Evaluations

Individuals’ characteristics and follow-up data collected during the ART procedures are described in Table 1. The mean ages of the two groups analyzed were 34.7 ± 3.16 and 34.4 ± 3.56; moreover, the BMI averages were 23.1 ± 2.9 kg/m^2^ and 23.0 ± 1.9, respectively. All the subjects were Caucasian, with smokers for no implantation and successful implantation constituting 30.62% and 28.17% respectively, male factors infertility constituting 32.65% and 61.97%, respectively, and those who underwent a long GnRH agonist protocol constituting 14.29% and 4.23% for each subgroup.

Moreover, we observed oocyte retrieval values of 4.85 ± 3.24 for no implantation and 5.9 ± 4.00 for successful implantation, as well embryos formed recorded as 2.40 ± 1.81 and 2.9 ± 1.63, respectively. The embryos transferred were 2.05 ± 1.25 and 2.38 ± 1.17 for each evaluated group, and the number of attempts were 1.17 ± 0.65 and 1.35 ± 0.56, as reported in Table 1. From statical comparison, none of the variables examined between the two groups were statistically significant.

### 3.2. Analysis of PTX3 Levels and Associated Signaling Pathway in the Follicular Fluid Among Women with and Without Successful Implantation

PTX3 is also produced by ovarian granulosa cells in response to local inflammatory stimuli as an earlier responsive pro-inflammatory protein than CRP [14,15]. Based on this evidence, tissue-specific PTX3 production from the follicles during IVF may serve as an early indicator of inflammation and high ovarian response and represent a reliable index of successful implantation outcome. Considering the sparse information available regarding PTX3 in IVF, we assessed its levels and its related main inflammatory pathway in the follicular fluid of women with a different history of IVF success through an ELISA assay.

**Figure 1:**
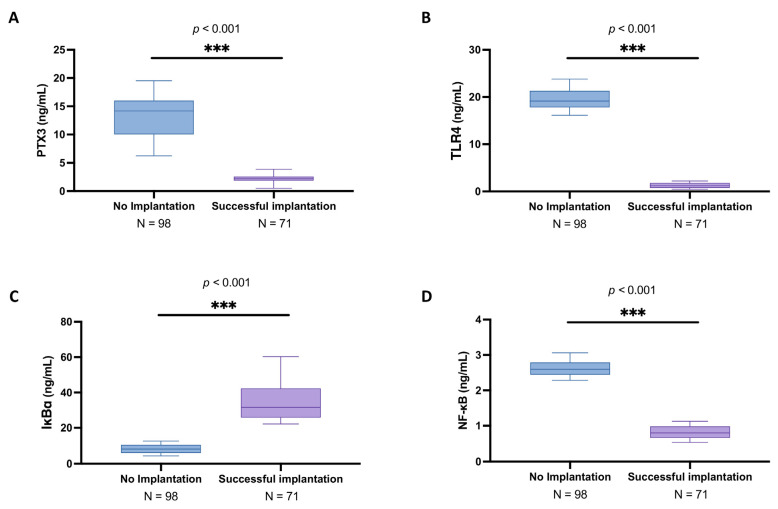
Follicular fluid PTX3 levels among no implantation women (*n* = 98) and women with successful implantation (*n* = 71). ELISA kits in follicular fluid to evaluate PTX3 (**A**), TLR4 (**B**), IkBα (**C**), and NF-κB levels (**D**). All data in the figure are shown as median with Interquartile Range (IQR). Data were not distributed normally. Statistical analysis was performed by using Mann–Whitney test. *** *p* < 0.001 vs. “No implantation”.

As shown in Figure 1A, in women in whom implantation did not occur, the PTX3 mean levels were 13.3 ± 3.35 ng/mL (median: 14.2; IQR: 10.1–16) compared to the successful implantation group (mean: 2.27 ± 0.55 ng/mL; median: 2.26; IQR: 1.86–2.57), highlighting a significant increase in the oocyte inflammatory state (*p* < 0.001). Similarly, TLR4 expression was significantly higher in the no implantation group (mean: 19.60 ± 2.18 ng/mL; median: 19.2; IQR: 17.8–21.3) compared to women that achieved successful implantation (mean: 1.25 ± 0.55 ng/mL; median: 1.18; IQR: 0.749–1.77) (Figure 1B; *p* < 0.001).

From the ELISA kits analyses, we also detected a significant degradation in IKBα levels in the women presenting no implantation (mean: 8.28 ± 2.57 ng/mL; median: 8.15; IQR: 5.99–10.6) compared to the peak of the other group (mean: 34.60 ± 10.70 ng/mL; median: 31.6; IQR: 25.7–42.3) (Figure 1C; *p* < 0.001). Consequently, NFκB expression was markedly increased in the no implantation group (mean: 2.63 ± 0.21 ng/mL; median: 2.6; IQR: 2.45–2.79) compared to successful implantation (mean: 0.82 ± 0.18 ng/mL; median: 0.8; IQR: 0.663–0.982) (Figure 1D; *p* < 0.001), further supporting the presence of an activated inflammatory profile in the follicular environment associated with unsuccessful IVF outcomes.

### 3.3. Analysis of Cytokines in the Follicular Fluid Among Women with and Without Successful Implantation

Local regulators of ovarian function are soluble factors of immunological origin such as cytokines [16]. Thus, we assessed the cytokines profile, in particular TNFα, IL-1β, and IL-6, in the follicular fluid samples by employing the ELISA method to verify local inflammation in women undergoing IVF.

**Figure 2:**
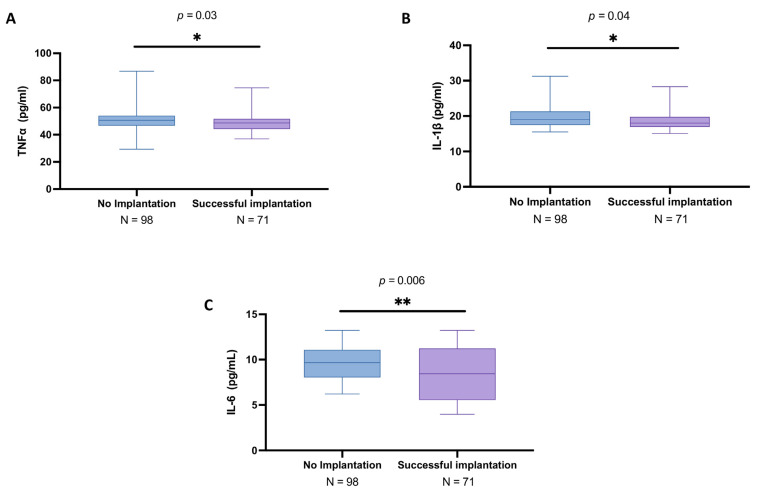
Follicular fluid levels of TNFα, IL-1β, and IL-6 among no implantation women (*n* = 98) and women with successful implantation (*n* = 71). ELISA assays executed for TNFα (**A**), IL-1β (**B**), and IL-6 (**C**). All data in the figure are shown as median with IQR. Data were not distributed normally. Statistical analysis was performed by using Mann–Whitney test. * *p* < 0.05 vs. “No implantation”; ** *p* < 0.01 vs. “No implantation”.

From our analyses, subjects with unsuccessful procedure results displayed slightly increased levels of TNFα (mean: 50.1 ± 7.84 pg/mL; median: 50.6; IQR: 46.7–54) compared to the other group (mean: 48.6 ± 6.35 pg/mL; median: 48.7; IQR: 44.2–51.7), leading to an appreciable statistically difference (*p* = 0.03), as indicated in Figure 2A.

Likewise, the results obtained from ELISA analysis revealed that IL-1β levels were slightly higher in the no implantation group (mean: 19.6 ± 2.96 pg/mL; median: 19.0; IQR: 17.4–21.3) compared to the women with successful IVF (mean: 18.9 ± 3.02 pg/mL; median: 18.0; IQR: 16.9–19.8) (*p* = 0.04), as reported in Figure 2B.

Moreover, the IL-6 levels showed significant differences between the two groups, with a mean concentration of 9.72 ± 2.00 pg/mL (median: 9.67; IQR: 8.05–11.1) in the no implantation group and mean of 8.42 ± 3.02 pg/mL (median: 8.44; IQR: 5.55–11.2) in the successful IVF group (*p* = 0.006), as illustrated in Figure 2C.

### 3.4. Subgroup Analysis of PTX3 Levels and Related Signaling Pathway in the Follicular Fluid of Women Under 30 Years Old with and Without Successful Implantation

We also analyzed women under 30 years old as a distinct subgroup due to the well-documented role of age in fertility outcomes. Research indicates that women under 30 tend to have higher fertility rates and more favorable responses to assisted reproductive technologies compared to older women. Therefore, this age group may have unique biological characteristics that could influence the success of implantation and the role of specific biomarkers. In this subset, the group with unsuccessful implantation included six participants, with a mean age of 28.2 ± 1.6 years. Among the participants, one out of six (16.67%) were smokers, and one out of six (16.67%) had male factor infertility. Additionally, one out of six (16.67%) participants underwent a long protocol for stimulation. In terms of clinical outcomes, the average number of oocytes retrieved was 4.0 ± 3.63, and the average number of embryos formed was 1.33 ± 1.21. Similarly, the number of embryos transferred into each participant was also 1.33 ± 1.21. On average, the group had 1.17 ± 0.983 attempts.

As well, the successful implantation group included 12 participants, with a mean age of 27.8 ± 1.91 years. Among the participants, 4 out of 12 (33.3%) were smokers, and 10 out of 12 (83.3%) had male factor infertility. None of the participants underwent a long protocol for stimulation. Regarding clinical outcomes, the average number of oocytes retrieved was 9.75 ± 2.30, and the average number of embryos formed was 3.67 ± 1.78. On average, 2.25 ± 1.22 embryos were transferred into each participant. In this group, the average number of attempts was 1.25 ± 0.452.

**Figure 3:**
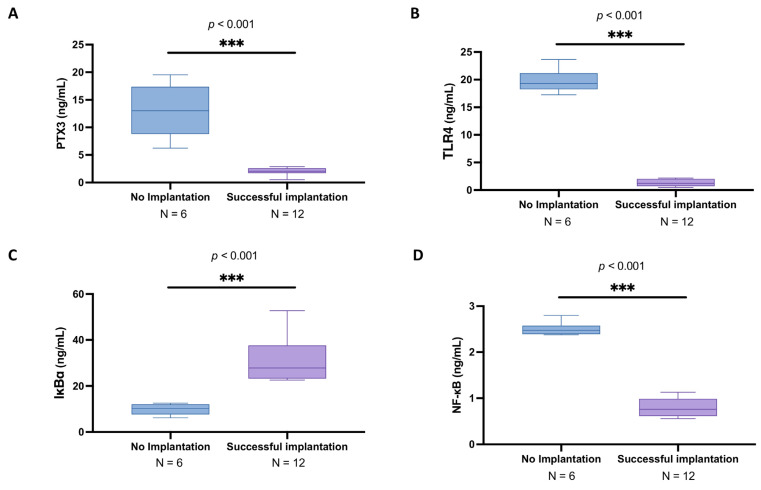
Follicular fluid PTX3, TLR4, IkBa, and NF-kB levels among under-30 no implantation women (*n* = 6) and women with successful implantation (*n* = 12). ELISA kits in follicular fluid to evaluate PTX3 (**A**), TLR4 (**B**), IkBα (**C**), and NF-κB levels (**D**). All data in the figure are shown as median with IQR. Due to the small sample size, statistical analysis was performed using Mann–Whitney test. *** *p* < 0.001 vs. “No implantation”.

In the subgroup analysis of women under 30 years old, the PTX3 levels in the follicular fluid still were significantly higher in the no implantation group (mean: 13.0 ± 4.79 ng/mL; median: 13.0; IQR: 8.83-17.4) compared to those with successful implantation (mean: 1.98 ± 0.76 ng/mL; median: 2.06; IQR: 1.70–2.59) (Figure 3A; *p* < 0.001).

As well, in this evaluation, TLR4 expression was upregulated in the no implantation group (mean: 19.7 ± 2.19 ng/mL; median: 19.3; IQR: 18.3–21.2) compared to the values of women presenting successful implantation (mean: 1.30 ± 0.64 ng/mL; median: 1.21; IQR: 0.688–2.04) (Figure 3B; *p* < 0.001).

Regarding the NF-kB pathway, a significant reduction in IKBα levels (mean: 9.84 ± 2.50 ng/mL; median: 10.2; IQR: 7.57–12.1) and upsurge in NFκB expression (mean: 2.50 ± 0.15 ng/mL; median: 2.47; IQR: 2.39–2.58) were observed in young women without effective implantation compared to the respective levels detected in the other group of IKBα (mean: 31.10 ± 9.30 ng/mL; median: 27.9; IQR: 23.2–37.6) and NFκB (mean: 0.79 ± 0.19 ng/mL; median: 0.758; IQR: 0.614–0.989) (Figure 3C and 3D, respectively; *p* < 0.001).

### 3.5. Subgroup Analysis of Cytokines in the Follicular Fluid of Women Under 30 Years Old with and Without Successful Implantation

We also assessed the cytokine profile, specifically TNFα, IL-1β, and IL-6, in follicular fluid samples from women under 30 years old undergoing IVF using the ELISA method.

**Figure 4:**
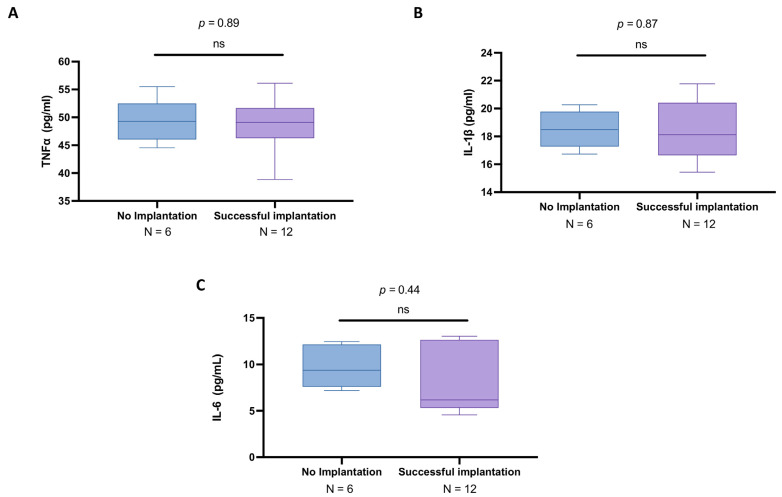
Cytokine levels of TNFα, IL-1β, and IL-6 in follicular fluid from younger women who did not achieve implantation (*n* = 6) and those with successful implantation (*n* = 12). ELISA assays were conducted to measure TNFα (**A**), IL-1β (**B**), and IL-6 (**C**). Results are expressed as median with IQR. Due to the small sample size, statistical comparisons were carried out using the Mann–Whitney test. ns: not significant vs. “No implantation”.

Here, our analysis revealed that subjects with unsuccessful procedure results exhibited similar TNFα levels (mean: 49.5 ± 3.85 pg/mL; median: 49.3; IQR: 46.0–52.5) compared to those with successful implantation (mean: 48.4 ± 4.96 pg/mL; median: 49.1; IQR: 46.3–51.7), resulting in no statistically significant difference (*p* = 0.89; Figure 4A).

Similarly, the IL-1β concentrations were more overlapped in the no implantation group (mean: 18.5 ± 1.32 pg/mL; median: 18.5; IQR: 17.3–19.8) than in the successful IVF group (mean: 18.4 ± 2.07 pg/mL; median: 18.1; IQR: 16.6–20.4) (*p* = 0.87), as displayed in Figure 4B.

The IL-6 levels demonstrated a more pronounced variation between the two groups, with a mean concentration of 9.69 ± 2.17 pg/mL (median: 9.36; IQR: 7.6–12.1) in women with failed implantation compared to the mean of 8.24 ± 3.58 pg/mL (median: 6.18; IQR: 5.31–12.6) in those who achieved implantation (*p* = 0.44), as illustrated in Figure 4C; however, statistical significance was also not achieved in this case.

### 3.6. Elevated PTX3 and NF-κB Pathway Alterations in Women over 30 with Unsuccessful Implantation

**Figure 5:**
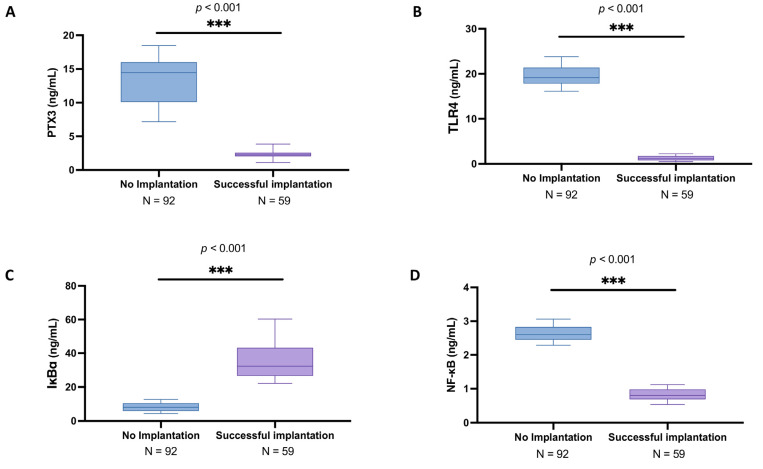
Follicular fluid levels of PTX3, TLR4, IKBα, and NF-κB in women over 30 with and without successful implantation. ELISA assays were conducted to measure PTX3 (**A**), TLR4 (**B**), IKBα (**C**), and NF-κB (**D**) concentrations in follicular fluid from women over 30 who experienced failed implantation (*n* = 92) and those with successful implantation (*n* = 59). Results are expressed as median with IQR. Non-normal distribution of data was detected. Statistical comparisons were performed using the Mann–Whitney test. *** *p* < 0.001 vs. “No implantation”.

In the subgroup analysis of women over 30 years old, the PTX3 levels in follicular fluid remained significantly elevated in the no implantation group (mean: 13.3 ± 3.27 ng/mL; median: 14.5; IQR: 10.1–16.0) compared to those who achieved successful implantation (mean: 2.33 ± 0.48 ng/mL; median: 2.27; IQR: 2.04–2.57) (Figure 5A; *p* < 0.001).

Analogously, TLR4 expression was markedly higher in women with failed implantation (mean: 19.6 ± 2.20 ng/mL; median: 19.2; IQR: 17.8–21.4) than in those with successful outcomes (mean: 1.24 ± 0.54 ng/mL; median: 1.18; IQR: 0.749–1.7) (Figure 5B; *p* < 0.001).

Regarding the NF-κB signaling pathway, a significant reduction in IKBα levels was observed in women who did not achieve implantation, with a mean concentration of 8.18 ± 2.56 ng/mL (median: 8.06; IQR: 5.91–10.4) compared to the mean of 35.3 ± 10.9 ng/mL (median: 32.4; IQR: 26.6–43.2) in the successful implantation group (Figure 5C; *p* < 0.001).

Additionally, NF-κB expression was significantly upregulated in the no implantation group (mean: 2.64 ± 0.22 ng/mL; median: 2.61; IQR: 2.45–2.83) compared to women who successfully implanted (mean: 0.83 ± 0.18 ng/mL; median: 0.800; IQR: 0.686–0.982) (Figure 5D; *p* < 0.001).

### 3.7. Cytokine Profile in Follicular Fluid of Women over 30 Years Old: A Comparison Between IVF Success and Failure

In this set of analyses, we also investigated the cytokine profile in the follicular fluid of women over 30 years old undergoing IVF, specifically focusing on TNFα, IL-1β, and IL-6 levels, measured using the ELISA method.

**Figure 6:**
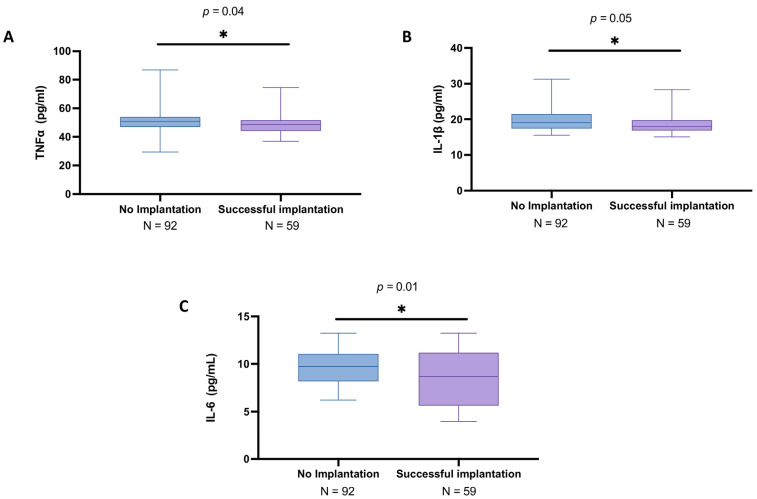
Cytokine levels of TNFα, IL-1β, and IL-6 were analyzed in follicular fluid from women over 30 years old who did not achieve implantation (*n* = 92) and those with successful implantation (*n* = 59). ELISA assays were used to measure TNFα (**A**), IL-1β (**B**), and IL-6 (**C**). Results are expressed as median with IQR. Non-normal distribution of data was found. Statistical analysis was performed using the Mann–Whitney test. * *p* < 0.05 vs. “No implantation”.

Our findings revealed that TNFα concentrations were significantly higher in women over 30 who experienced implantation failure (mean: 50.2 ± 8.04 pg/mL; median: 50.8; IQR: 46.8–54.1) compared to those with successful implantation (mean: 48.7 ± 6.63 pg/mL; median: 48.7; IQR: 44.2–51.7), with a slight statistically significant difference observed (*p* = 0.04) (Figure 6A).

As well, the IL-1β levels showed a significant difference, with a mean concentration of 19.7 ± 3.03 pg/mL (median: 19.1; IQR: 17.4–21.5) in the failure group and a mean of 19.0 ± 3.19 pg/mL (median: 18.0; IQR: 16.9–19.8) in the successful implantation group (*p* = 0.05) (Figure 6B).

The IL-6 levels also demonstrated a significant variation, with a mean concentration of 9.72 ± 2.00 pg/mL (median: 9.75; IQR: 8.2–11.1) in the failed implantation group compared to a mean of 8.46 ± 2.93 pg/mL (median: 8.72; IQR: 5.64–11.2) in those with successful implantation (*p* = 0.01) (Figure 6C).

### 3.8. Correlation Analysis of PTX3 and Cytokines Relating to Implantation Rate in Women with and Without Successful Implantation

A measure that is frequently used to summarize and report on ART outcomes is the combination of the implantation rate and the pregnancy rate [17]. The implantation rate is determined by the total number of embryos transplanted and the total number of gestational sacs product [17].

**Figure 7:**
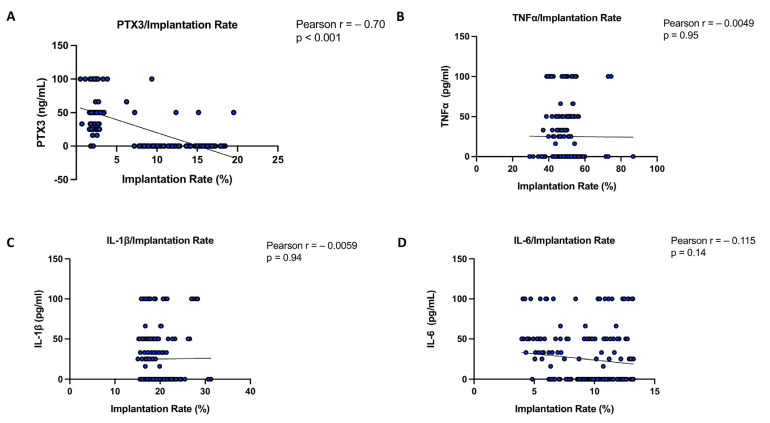
PTX3 and cytokines correlation with implantation rate. Only a *p*-value of less than 0.05 was considered significant.

Therefore, by using Pearson analysis, the association between PTX3 protein levels and implantation rate (expressed as a percentage) was also evaluated and compared with cytokines. From this evaluation, the Pearson correlation coefficient r = −0.70 for PTX3 indicates a strong negative correlation with implantation rate percentage, with *p* < 0.001, confirming statistical significance. In contrast, the cytokines TNFα (r = −0.0049; *p* = 0.95), IL-1β (r = −0.0059; *p* = 0.94), and IL-6 (r = −0.115; *p* = 0.14) do not show any significant correlation with the implantation rate, indicating that variations in their levels are not associated with changes in implantation success (Figure 7).

## 4. Discussion

The inflammatory state in the follicular fluid and its effects on IVF outcomes have been of great interest in recent years [10]. Inflammation has important physiological implications in ovarian folliculogenesis and ovulation [18]. Nevertheless, a growing body of research indicates that aberrant inflammation might modify the dynamics of normal ovarian follicles, leading to anovulation, decreased oocyte quality, and consequent infertility [18].

It has been suggested that the follicular microenvironment plays a significant role in the growth and maturation of oocytes [19]. Indeed, the development of early embryos, fertilization, follicular wall rupture, and oocyte maturation are all impacted by the amounts of inflammatory mediators in the follicular fluid [20,21].

For instance, TNFα, IL-1β, IL-2, and IL-6 are all cytokines that control several molecules involved in the immunological and inflammatory responses [22]. Since few research studies have examined these inflammatory markers in ART settings, their levels and function in the follicular fluid still need to be fully determined.

In this study, our attention focused on the follicular fluid levels of PTX3 in comparison to other well-known inflammatory markers. PTX3 is a well-known effector molecule belonging to the humoral arm of innate immunity, and it is rapidly produced and released by several cell types in response to primary inflammatory signals [23]. PTX3 is a downstream target gene of GDF9 and a member of the transforming growth factor-β (TGF-β) superfamily, playing an important role in promoting cumulus expansion, which is a critical process [24]. In addition, PTX3 represents a matrix protein that interacts with hyaluronan in the expanded cumulus matrix [25]. Moreover, it has been recognized that PTX3 plays a non-redundant role in innate immunity by opsonizing selected pathogens and in female fertility. Unlike short pentraxin or CRP (that is predominantly produced in the liver), PTX3 is produced in the local site of the inflammation [26], including follicle cells, and it is essential for the organization of the cumulus oophorus extracellular matrix [15].

Other studies described that neither the PTX3 transcript level nor the PTX3 protein concentration in the follicular fluid exhibited a significant association with the oocyte quality and morphological grade of the embryo; as well, higher PTX3 levels in women without ART success could represent an excessive maternal low inflammatory state that undermines embryo implantation [23].

Therefore, looking for novel potential biomarkers in association with implantation success in the ART process, we checked the PTX3 levels in comparison with the best-known cytokines in collected follicular fluids. ART procedures were performed only after GnRH agonist cycles in ovarian stimulation of non-obese young women. No statistical differences between the two groups in the study were observed regarding the collected clinical information and fertility-related parameters.

Elevated PTX3 levels in the follicular fluid of women could likely be associated with altered immune responses and poor oocyte quality. One possible mechanism linking PTX3 to impaired fertility involves the TLR4/NF-κB signaling pathway. TLR4 activation by endogenous danger signals in the ovarian follicle triggers NF-κB translocation, leading to the upregulation of pro-inflammatory species and PTX3 expression as well. This inflammatory cascade may contribute to an altered follicular milieu, disrupting oocyte maturation and reducing implantation potential. Thus, increased PTX3 levels in follicular fluid could serve as both a marker and a mediator of an excessive inflammatory response in women during ART procedures, further reinforcing the link between innate immunity dysregulation and reproductive failure.

In the present study, the PTX3 levels in follicular fluid among women with unsuccessful implantation were almost 6-fold higher compared to the positive implantation group, proving to be a more promising biomarker of the worst ART outcome. Additionally, we observed an upregulation of TLR4 and NF-κB, suggesting a heightened inflammatory response in the follicular environment. This increase in TLR4 expression may contribute to the activation of the NF-κB pathway, leading to the production of pro-inflammatory mediators that could negatively impact oocyte quality and implantation potential. Notably, these findings were further confirmed in subgroup analyses stratified by age, with consistent results observed in both women over 30 years old and those under 30. This supports the hypothesis that dysregulation of the TLR4/NF-κB axis and elevated PTX3 levels are closely linked to poor reproductive outcomes, regardless of age.

Cytokines in follicular fluid play a crucial role in regulating ovarian function, oocyte maturation, and embryo implantation, since they mediate immune responses, modulate inflammation, and influence follicular development through complex signaling pathways [27].

In particular, TNFα is a cytokine that affects many kinds of cells in pleiotropic ways [28]. It is generated by leukocytes, especially macrophages, as well as a few other cells, such as smooth muscle cells, that are not normally associated with the immune system [28].

Even though TNFα was first linked to systemic and local inflammation, more recent research has revealed that this cytokine is present in the ovarian cells and follicular fluid of several species, including humans, rats, and domestic animals [29].

In some of these species, TNFα is produced by oocytes, granulosa cells, thecal cells, luteal cells, endothelial cells, and macrophages [29]. In particular, luteal regression, ovulation, and follicular development are the three ovarian processes that control the expression of ovarian TNFα [29]. Similarly, as a member of the IL-1 family, IL-1β has pro-inflammatory properties [30].

The first trimester of pregnancy is when cytotrophoblast cells primarily release IL-1β at high levels; after that, its production is reduced [31,32].

Scientific evidence has indicated that IL-1β can also stimulate cytotrophoblasts’ production of matrix metalloproteinase-9, which is a crucial molecule that mediates trophoblast invasion [33]. In addition, B3 integrin, a recognized implantation marker, is also expressed more when IL-1β is present [33].

Furthermore, in vivo investigations on mice have shown a connection between IL-1 components and the development of implantation and pregnancy, indicating that the endometrium expresses both the IL-1β and the IL-1 (IL-1R) receptors [34]. Moreover, IL-1R, IL-1β, and IL-1Ra are all present in embryos, and in particular, the release of IL-1 family components from the embryos is shown to be dependent on co-culture with maternal endometrial components [35]. Additionally, this expression of IL-1R, IL-1β, and IL-1Ra is both temporally and spatially related to embryonic implantation onto the endometrium [36,37,38].

In this context, IL-6 also represents a key pro-inflammatory cytokine in follicular fluid involved in ovarian follicle development, oocyte maturation, and corpus luteum function [39]. It plays a dual role, promoting follicular growth at physiological levels but contributing to an inflammatory microenvironment when excessively elevated [39]. Increased IL-6 levels in follicular fluid have been associated with oxidative stress, impaired granulosa cell function, and poor oocyte quality, potentially leading to lower fertilization and implantation rates. Furthermore, dysregulated IL-6 signaling is often observed in conditions like polycystic ovary syndrome (PCOS) and endometriosis, linking chronic inflammation to female infertility [10,40].

Overall, it can be assumed that cytokines, especially triggering a Th1-type immune response, may be largely involved in infertility and recurrent spontaneous abortions [41].

Thus, since it is well known that proinflammatory TNFα, IL-1β, and IL-6 are present in the follicular fluid, have pleiotropic effects on multiple reproductive system sites, and that gonadotropins and pituitary hormones can regulate their production or secretion, we examined potential changes in their levels in the current study.

Our results showed that the levels of IL-1β and TNFα in the follicular fluid of patients with successful implantation, compared to the no implantation group, indicated a significantly higher inflammatory response with statistical significance. This confirms that a pro-inflammatory environment within the follicle may play a critical role in implantation success, potentially influencing oocyte quality and endometrial receptivity. However, in the subgroup analysis based on age, the cytokine levels were not significantly different between the two groups in women with a mean age below 30 years. In contrast, in women over 30, the IL-1β and TNFα levels were slightly higher in the no implantation group.

A possible explanation for this discrepancy could be that younger women have a greater ovarian reserve and a more resilient follicular microenvironment, allowing them to compensate for mild inflammatory changes without significantly affecting implantation success. In contrast, older women may experience a decline in ovarian function, including lower levels of hormones, reduced estrogen production, and altered progesterone signaling, all of which contribute to decreased follicular quality and endometrial receptivity. These hormonal changes, combined with increased susceptibility to inflammation and oxidative stress, may exacerbate the negative effects of elevated IL-1β and TNFα, ultimately impairing implantation success in women over 30, although these assumptions will need to be confirmed through more in-depth future investigations.

In agreement with previous scientific studies [1,42], our results support the upstream of PTX3 in subjects with no implantation compared to women with a positive IVF outcome.

These data were also confirmed by the correlation analysis, in which the results indicated a strong negative correlation between the PTX3 levels in follicular fluid and implantation rate, while TNFα, IL-1β, and IL-6 showed no significant association with implantation success. A plausible explanation for these findings lies in the different roles these molecules play in the follicular microenvironment and subsequent implantation process. Indeed, PTX3 is involved in inflammation, tissue remodeling, and extracellular matrix organization, all of which are crucial for oocyte quality and embryo implantation. High PTX3 levels in follicular fluid may reflect an altered inflammatory state or suboptimal follicular environment, potentially affecting oocyte competence and subsequent endometrial receptivity and ultimately leading to a lower implantation rate. Elevated PTX3 has been linked to oxidative stress and inflammatory responses [43], which could impair embryo development or implantation capacity.

Conversely, TNFα, IL-1β, and IL-6 are well-known inflammatory cytokines, but their lack of correlation with implantation rate suggests that their presence in follicular fluid does not directly impact embryo implantation. These cytokines may play localized roles in follicular development, ovulation, or early embryonic stages, without exerting a long-term effect on implantation. Additionally, their influence may be more dependent on local concentration gradients, receptor interactions, or systemic immune–endocrine balance rather than absolute levels in follicular fluid.

Overall, these findings suggest that PTX3 in follicular fluid may serve as a potential biomarker of oocyte competence and implantation success, whereas TNFα, IL-1β, and IL-6 levels in follicular fluid do not appear to have a direct predictive role in implantation outcomes.

Likewise, Freis et al. reported that the serum levels of PTX3 were lower in women with subsequent ongoing pregnancies, confirming, similarly, the negative impact of elevated systemic PTX3 on implantation. In addition, the authors speculate that implantation failure can be associated with an increase in Th1-dependent cytokines, such as TNFα, that seem to upregulate PTX3. This process determines unsuccessful embryo implantation that is probably linked to inflammatory and immune changes [1], but in our preliminary results, the IL-1β and TNFα levels were comparable between the two groups and thus were not upregulated in the follicular fluid of women with no implantation.

However, despite these remarkable findings and the novelty of presenting PTX3 as a useful marker in this clinical context, which represents the greatest strength of this pilot study, some limitations need to be taken into consideration. Surely, the main limitation of the study is the small sample size, which needs validation in a larger population.

Particularly, in the subgroup analysis comprising women under the age of 30, the limited sample size reduces the statistical power of the analysis and makes it difficult to draw firm conclusions. While we acknowledge this limitation, the decision to include this subgroup was driven by the desire to explore potential differences in a clinically relevant context, despite the small sample. Thus, we emphasize that the findings from this subgroup analysis should be interpreted with caution and need to be confirmed in future studies with large cohorts; this will enhance the generalizability and statistical power of the findings.

The use of an arbitrary age cutoff (<30 years) for subgroup analysis could introduce a level of bias, as fertility parameters can vary not only between age groups but also within the same age group. Although we justified the cutoff based on established guidelines in reproductive medicine, future studies could benefit from using a more detailed age stratification to better characterize the heterogeneity in fertility dynamics across distinct subpopulations.

Moreover, all our participants were Caucasian, so our preliminary results may not apply to other ethnicities. Furthermore, we did not evaluate the levels of these factors over time. New approaches, such as the study circulating of free DNA and/or RNA in follicular fluid, will be able to confirm the level of expression of the *PTX3* gene and other factors, which may be associated with a greater possibility of success in ART [44,45]. However, this peculiar context, which sees follicular fluid and pregnancy success as the specific pilot outcomes of the study, requires close collaboration with ART centers and time for follow-up of couples.

In summary, the most relevant and significant preliminary clinical data we obtained on the follicular fluid are increases in PTX3 levels, influencing the ovarian microenvironment, which could be associated with an increased risk of no implantation and a low rate of IVF success. As a result, the characterization of the follicular fluid integrating genetics, biochemical, and expression analysis concerning complex clinical conditions, such as infertility, may lead to the discovery of new reliable markers that can improve pregnancy rates in clinical practice [46,47].

The advancement of contemporary reproductive care medicine also depends on the assessment of novel diagnostic biomarkers that can distinguish successful outcomes in patients receiving ART.

In this regard, some investigations showed that PTX3 is a novel biomarker to take into account in routine analyses, even though the diagnostic advantage of PTX3 over CRP or other inflammatory markers is still under debate. Hamed and colleagues, for example, investigated the usefulness of PTX3 in septic patients and found that it was as useful as IL-6 in diagnosing sepsis and septic shock in the first week of intensive care therapy [48]. Likewise, the results of our research advanced the accuracy of PTX3 over TNFα and IL-1b in predicting an increased risk of implantation failure.

Certainly, PTX3 also represents a promising therapeutic target for several inflammatory-related disorders, including clinical concerns associated with reproduction, because of its essential modulatory roles on the inflammation cascade and humoral innate immunity.

Future clinical investigations aimed to understand the multiple mechanisms of PTX3 in the ovary, as well as the knowledge of current treatment strategies to target this process, may pave the way to their application in the clinical field.

## 5. Conclusions

To increase the knowledge about a possible assessment of implantation, our primary outcome suggests that PTX3 could represent a possible biomarker for ART success, suggesting its useful measurement in women with no explained successful implantation.

Taken as a whole, our preliminary findings may contribute significantly to the identification of new biomarkers, representing an important add-on in the field of ART, IVF, and human fertility research.

Furthermore, considering the importance of PTX3 in inducing immune–inflammatory responses in follicular environments, it can be useful to expand our cohort and test the PTX3 levels in follicular fluids or other biological fluids in clinical settings. We further speculate that inhibiting the PTX3 pathway can represent a strategy to manage the inflammatory response in several clinical conditions, such as reproductive dysfunction. This concept could lead to fewer stimulation cycles for women, minimizing the risk of possible side effects and psychological or/and emotional stress. To improve the consistency of our outcomes, further studies are underway to validate and expand the conclusions of these preliminary results.

## Figures and Tables

**Table 1:** Characteristics of included subjects. All the values are reported as mean ± SD.

	No Implantation (*n* = 98)	Successful Implantation (*n* = 71)	*p*-Value (*t*-Test)
Mean age	34.7 ± 3.16	34.4 ± 3.56	0.56
BMI (kg/m^2^)	23.1 ± 2.9	23.0 ± 1.9	0.80
Smokers	30/98 (30.61%)	20/71 (28.17%)	
Male factor	32/98 (32.65%)	44/71 (61.97%)	
GnRH long protocol	14/98 (14.29%)	3/71 (4.23%)	
Oocytes retrieved	4.85 ± 3.24	5.9 ± 4.00	0.06
Embryos formed	2.40 ± 1.81	2.9 ± 1.63	0.07
Embryos transferred into each patient	2.05 ± 1.25	2.38 ± 1.17	0.08
Number of attempts	1.17 ± 0.65	1.35 ± 0.56	0.06

## Data Availability

All the results were included in this study and are available to obtain from the corresponding author’s address.

## Abbreviations

ART	Artificial reproductive treatment
PTX3	Pentraxin 3
IVF	In vitro fertilization
TNFα	Tumor necrosis factor alpha
IL	Interleukin
CRP	C-reactive protein
SAP	Serum amyloid P component
TNFαIP6	Tumor necrosis factor a-induced protein 6
ELISA	Enzyme-linked immunosorbent assays
AMH	Anti-Müllerian Hormone
AFC	Antral follicle count
FSH	Follicle stimulating hormone
GnRH	Gonadotropin-releasing hormone
hCG	Human chorionic gonadotropin
ICSI	Intracytoplasmic sperm injection
BMI	Body mass index
SD	Standard deviation
